# Culturally Competent in Medical Education – European Medical Teachers’ Self-Reported Preparedness and Training Needs to Teach Cultural Competence Topics and to Teach a Diverse Class

**DOI:** 10.15694/mep.2019.000098.1

**Published:** 2019-04-26

**Authors:** Katja Lanting, Nisha Dogra, Kristin Hendrickx, Yoga Nathan, Judith Sim, Jeanine Suurmond

**Affiliations:** 1 Department of Public Health; 2 Greenwood Institute of Child Health; 3 Department of Primary and Interdisciplinary Care; 4 Graduate Entry Medical School; 5 Biomedical Teaching Organisation (BMTO)

**Keywords:** medical education, diversity, training needs, cultural competence, diverse class

## Abstract

This article was migrated. The article was marked as recommended.

Background

Health inequalities related to culture and ethnicity may be reduced by training future health care providers.Medical teachers therefore also need to be culturally competent. The aim of this study was to assess medical teachers’ preparedness and their training needs to teach cultural competence topics and to teach a diverse class.

Methods

A link to an online survey was sent to medical teachers of eleven European institutions. Results were analysed through descriptive analysis and answers to open-ended questions were analysed using qualitative analysis.

Results

968 respondents were included. The majority of respondents felt it was important that cultural competence topics should be incorporated into the medical curriculum. Assessment of skills in cultural competence was found important as well.

Over 60% of all respondents reported to be somewhat or very prepared to teach cultural competence topics like migrant health and disparities. Most respondents felt somewhat or very prepared to teach a diverse class. A high interest in training was expressed on teaching cultural competence topics, specifically on communication-related topics.

Conclusion

This study emphasizes the importance of incorporating cultural issues into the medical curriculum and to train medical teachers according to their needs.

## Introduction

Due to global migration, Europe constitutes increasingly more culturally and ethnically diverse populations. Generally, migrants and ethnic minorities are less physically and mentally healthy than the majority population. For example, migrants and ethnic minorities in Europe generally are over-represented in cardiovascular diseases, diabetes and obesity (Meeks *et al*., 2016), and generally perceive themselves as less healthy than the majority population ( Nielsen *et al*., 2010).

Additionally, migrants and ethnic minorities do not receive the same quality of care as patients from majority populations. Studies on for example, access to home care ( Suurmond *et al*., 2016), patient safety ( van Rosse *et al*., 2015), prognosis after an acute myocardial infarction hospitalisation ( Oeffelen, 2014) and optimal treatment ( Green *et al*., 2007), show that health care for migrants and ethnic minorities is less optimal when compared to majority populations. It is believed that communication barriers, including language barriers, play a role, as well as cultural barriers and bias of health care providers. Health care providers generally communicate less effectively with migrants and ethnic minorities (Paternotte, 2015; Meeuwesen, 2006) and show less affective behaviours. While it is generally agreed that cultural competence training can result in better access to and increased quality of care for migrants and ethnic minorities ( Horvat, 2014; Tuong, 2014; Beach 2005; Chiarenza 2018), cultural and ethnic diversity is not an integrated part of medical education and cultural diversity training is an under-represented topic in the medical curriculum ( Sorensen, 2017a, Paternotte *et al*., 2014; Smedley *et al*., 2003; Van Wieringen, 2003).

A study on the status of teaching diversity in North-America (US and Canada) ( Dogra *et al*., 2010) similarly revealed a lack of conceptual clarity, and pointed at the fragmented and variable programs to teach cultural diversity. Faculty and staff support and development with regard to teaching diversity, and ambivalence from both staff and students about the relevance continue to be a challenge as well.

Many organisations concerned with quality of and equity in health care emphasised the need for training of students and health professionals in cultural competence, including the HPH Task Force on Migration, Equity & Diversity; Joint Commission Resources; the Council of Europe; WHO regional office Europe; and the International Organisation for Migration. All urge for more attention for cultural competence topics in medical education.

Cultural competence in medical education is commonly defined as a set of attitudes, knowledge and skills that are necessary for care providers to effectively interact with culturally and ethnically diverse patient populations ( Dogra *et al*., 2016; Seeleman *et al*., 2009; Betancourt, 2003). It is ultimately meant as the ability to provide effective health care services taking into consideration the individual’s gender, sexual orientation, disability, age and religious, spiritual and cultural beliefs ( Council of Europe, 2006). Besides analogue approaches, such as equity ( Whitehead *et al*., 2006), superdiversity ( Vertovec, 2007) and intersectionality ( Hankivsky *et al*., 2014), cultural competence is a frequently adopted framework for training programmes ( Chiarenza *et al*., 2018).

To provide medical students with cultural competencies, medical teachers also need to be equipped with competencies to teach these issues to students. We, however, do not know what the state of the art is in Europe.

To fill this gap, the present study was performed investigating European medical teachers’ training needs towards teaching cultural competence topics and teaching a diverse class, involving medical schools in eleven European countries. More specific goals of this study were to identify medical teachers’ opinions on cultural competence topics in medical education, their preparedness to teach cultural competence topics and teaching a diverse class and their training needs regarding teaching cultural competence topics and teaching a diverse class.

This study was part of the Culturally Competent in Medical Education (C2ME) project (2013-2015), which aimed to contribute to the integration of cultural competence teaching in undergraduate medical curricula in Europe (see Suurmond *et al*., 2015; Sorensen *et al*, 2017a; Sorensen *et al*., 2017b; Hudelson *et al*., 2016; Hordijk, 2018). C2ME was financially supported by Erasmus Life Long Learning Program, with medical schools in 11 European countries participating.

## Methods

A web-based survey was jointly developed by the team at the Academic Medical Center/University of Amsterdam with input from all the C2ME partners. The items were closely discussed and agreed upon. A small pilot was done among four external people, who were not involved in the C2ME project to check for relevance and clarity.

The questionnaire consisted of 41 items. The items were grouped under five headings: 1. Opinions about cultural competence in health care and medical education (nine items); 2. Preparedness to teach cultural competence topics to medical students (ten items); 3. Preparedness to teach a diverse classroom (four items); 4. Interest in receiving training (Training needs) on teaching cultural competence topics (ten items); 5. Interest in receiving training on teaching a diverse classroom (four items). The 41 five-point Likert-scale questions were followed by two open-ended questions and eight questions to collect demographic information. A link to the online English-only survey, built using the programme Survey Gizmo, was then sent to the coordinator of each of the 11 institutions involved in the C2ME project. Each coordinator forwarded the survey to the relevant staff at their institution. The selected medical teachers were invited to participate (as a survey respondent) by email. In total, the survey was sent to 5577 medical teachers between January and February 2015. The invitation to participate was accompanied by a cover letter which was translated by each institution to their own language.

After two weeks a reminder was sent to all respondents with a second reminder a further two weeks later. Data collection was stopped a week after the second reminder. The study was approved by the Dutch NVMO Ethical Review Board (NERB, file number 324).The ethical principles for medical research involving human subjects as laid down in the Declaration of Helsinki and adopted by the World Medical Association were followed. Codes were used to designate the respondents to guarantee their anonymity. Each respondent was adequately informed of the aims and methods of the study, and a priori informed consent was obtained from the respondents for their participation in this study.

The results were analysed through descriptive calculations using SPSS 23. Answers to open-ended questions were analysed using qualitative analysis (Miles and Huberman, 2014). Coding was done by reading the text line by line, and a paraphrase or label (a code) was applied to those fragments that were relevant. Open coding (coding that might be relevant from as many different perspectives as possible) as well as some predefined codes (based on the constructs of the questionnaire, e.g. ‘preparedness’ or ‘training needs’) were used. This set of codes was the thematic framework, which was applied to all the texts. A chart was devised with headings and subheadings and for each respondent entries were made.

The number of selected medical teachers differed per institution, for example, the partner in Copenhagen sent an invitation to 1400 medical educators whereas the partner in Pécs sent it to 600 and the partner in Edinburgh to 202. The response rate per institution therefore varied as well (see table I).

**Table I.** Participants per institution (responded, proportional response, invited and response rate)

*Name of institution*		*Participants*	*Participants in %*	*Invited People*	*Response rate (%)*
	1 University of Geneva	108	12.9	457	23.6
	2 University of Limerick	29	3.5	300	9.7
	3 University of Leicester	20	2.4	143	14.0
	4 University of Edinburgh	33	3.9	202	16.3
	5 University of Giessen	14	1.7	220	6.4
	6 VU University Medical Center Amsterdam	68	8.1	196	34.7
	7 University of Copenhagen	199	23.7	1400	14.2
	8 University of Antwerp	44	5.2	467	9.4
	9 AMC/University of Amsterdam	131	15.6	739	17.7
	10 University of Bergen or Oslo and Akershus University College	23	2.7	175	13.1
	11 University of Pécs	100	11.9	600	16.7
	12 University of Sevilla	71	8.5	678	10.5
	Total	840	100.0	5577	15.1 *
	System Missing	128			
**Total**		**968**			

* The overall response was 968. As seen in table I, 128 of these 968 people did not report which institution they were affiliated with.

## Results/Analysis

### Respondent Characteristics

968 respondents completed the survey, which is a response rate of 17.4%. About half of the group was female (52.9%) and 63.2% of the respondents was aged between 41 and 60 (see table II). The people involved in teaching cultural competence to medical students in this sample were for the most part leaders or senior staff (nearly 74%).

Most respondents were medical doctors (MD), 69.5%. However, the sample included nurses, biomedical scientists, public health professionals and social scientists (6.8% - 9.2%). Many respondents participated in teaching clinical skills in simulation context (56.6%), basic/biomedical skills (36.1%), and ward and clinic based teaching (30.7%). 17.4% had completed previous training in cultural competence topics. 12% of the respondents indicated that they belonged to a cultural or ethnic minority group in their country of residence. Within this specific group, more respondents (28.4%) had completed a training in cultural competence topics, compared to 17.4% of the total sample.

**Table II.** Participants characteristics

	N	%
**Age** (n=873)		
<30 years	32	3.7
31-40 years	179	20.5
41-50 years	268	30.7
51-60 years	284	32.5
>61 years	110	12.6
**Gender** (n=867)		
Male	406	46.6
Female	461	52.9
**Self-definition cultural or ethnic minority status** (n=869)		
Yes	107	12.3
No	762	87.7
**Position** (n=869)		
Head/leading	259	29.8
Senior staff member	469	54.0
Junior staff member	141	16.2
**Completed training in cultural competence topics** (n=860)		
Yes	150	17.4
No	710	82.6
**Professional background** (n=888)		
MD	617	69.5
Nurse	67	7.5
Biomedical scientist	82	9.2
Public Health	60	6.8
Social scientist	65	7.3
More than one professional background	87	9.8

### Opinions About Cultural Competence in Medical Education

Nearly 90% of all respondents agreed or strongly agreed that ‘Medical education should include training about cultural issues’ (see table III). In the open text it was mentioned that vertical integration of cultural diversity subjects in the curriculum is needed. Several respondents noted that not everyone in their environment is aware of the importance and the relevance of teaching cultural competence.

Various suggestions were made about how to integrate cultural competence in the curriculum: *Make it a part of clinical education. With doctors giving their examples of integration. Importantly, not [design it] as a theoretical course.”* Another respondent wrote: *“Data or at least examples of how cultural competence has an impact on both practice and (ideally) outcomes would be helpful.”* Another wrote: *“While important to incorporate into ongoing educational activities it needs to be recognized and covered as a distinct module in the curriculum.”* And yet another wrote: *“Disperse material, cases, news stories et cetera that illustrate the importance of this issue and make it relevant to them - even those who might not automatically meet it in their daily practice. And training - you do not address this if you don’t feel competent.”*

73.4% of respondents agreed or strongly agreed that ‘During medical school students must be assessed for skills in cultural competence’. A respondent reported: *“It is a crucial transverse topic: (...) making sure there are questions in the examinations, have training for medical teachers about this topic”.*

When asked about cultural competence in one’s own practice, the vast majority of respondents agreed or strongly agreed (92.1%) that ‘ *Patients* look at health problems through their own cultural lens’. When asked, ‘ *Care providers* look at health problems through their own cultural lens’, a smaller proportion (74.1%) agreed or strongly agreed. An even smaller proportion (68.1%) agreed or strongly agreed to the item ‘I view the health system through my own cultural lens’.

37% neither disagreed nor agreed regarding ‘I recognize when my reactions are based on stereotypes’. Slightly more than half (54%) agreed or strongly agreed that they recognized it when one’s reactions were based on stereotypes, yet only 9% disagreed or strongly disagreed. Many respondents suggested that training may help, for example this one: “Making them aware of their own (differences in) thoughts and behaviors in relation to minority/majority patients.”

**Table III.** Teachers’ opinions about cultural competence in health care and medical education Reported in percentage (n)

	Strongly disagree	Disagree	Neither disagree nor agree	Agree	Strongly agree
Patients look at health problems through their own cultural lens *(missing=1)*	.9% (9)	.8% (8)	6.1% (59)	57.7% (558)	34.4% (333)
Care providers look at health problems through their own cultural lens *(missing=3)*	1.0% (10)	6.8% (66)	18.0% (174)	60.2% (581)	13.9% (134)
I view the health system through my own cultural lens *(missing=4)*	2.2% (21)	10.6% (102)	19.2% (185)	57.9% (558)	10.2% (98)
Medical education should include training about cultural issues *(missing=6)*	.5% (5)	1.8% (17)	9.3% (89)	51.5% (495)	37.0% (356)
I recognize when my reactions are based on stereotypes *(missing=0)*	.4% (4)	8.6% (83)	37.0% (358)	47.8% (463)	6.2% (60)
During medical school students must be assessed for skills in cultural competence *(missing=6)*	1.1% (11)	5.0% (48)	20.5% (197)	50.0% (481)	23.4% (225)

### Preparedness to Teach Cultural Competence Topics

Respondents were relatively well prepared to teach ‘How to explore aspects of the patient’s social context that could have an impact on care’ (64.9%). More than half of the respondents (54.2%) answered that they felt somewhat or very prepared to teach about prejudice and discrimination in health care. Respondents felt less prepared to teach on ‘How to address conflict when there are different cultural views between the care provider and the patient’, less than half of all respondents reported being somewhat or very prepared (46.2%). About half of the respondents felt somewhat prepared or very prepared teaching ‘How to explore students’ perspectives and reflect on how these may influence their future practice’; teaching ‘Disparities in health and health care’; and teaching ‘Migrant health’ (47.5% to 50.1% was found in ascending order).

A respondent said: *“Teachers should become more sensitive to this topic in general. The purpose and need for cultural competence should be made very clear”. Another reported: “As a student I would have appreciate it to learn more about different cultural backgrounds and their impact on medical topics. Now I am teaching students and I sometimes feel a bit unprepared when it comes to language problems (or maybe other problems of understanding).”*

**Table IV.** Teachers’ opinion of own preparedness to teach cultural competence topics. Reported in percentage (n)

	Very unprepared	Somewhat unprepared	Neither unprepared nor prepared	Somewhat prepared	Very prepared
How to explore the patient’s cultural and religious beliefs that could have an impact on their care *(missing=2)*	7.9% (76)	20.0% (193)	21.2% (205)	41.2% (398)	9.7% (94)
How to explore aspects of the patient’s social context that could have an impact on care *(missing=4)*	3.6% (35)	12.9% (124)	18.6% (179)	47.5% (458)	17.4% (168)
How to work effectively with interpreters *(missing=10)*	10.1% (97)	17.4% (167)	21.7% (208)	34.6% (331)	16.2% (155)
About prejudice and discrimination in health care *(missing=8)*	4.2% (40)	16.0% (154)	25.6% (246)	41.3% (396)	12.9% (124)
About migrant health (epidemiology, social determinants, access and barriers to care, etc.) *(missing=12)*	9.3% (89)	18.8% (180)	21.8% (208)	40.3% (385)	9.8% (94)
How to address conflict when there are different cultural views between the care provider and the patient *(missing=17)*	8.0% (76)	23.1% (220)	22.7% (216)	36.7% (349)	9.5% (90)
How to prepare students to adapt their communication style to respond to the patient’s needs and capabilities *(missing=13)*	6.2% (59)	18.2% (174)	20.0% (191)	43.5% (415)	12.1% (116)

### Preparedness to Teach a Diverse Classroom

63.4% reported being somewhat prepared or very prepared to ‘Teach students from a range of diverse cultural and religious backgrounds’. When asked about preparedness to ‘Examine one’s own cultural biases that may influence one’s behaviour as a teacher’, 63.9% reported being very or somewhat prepared, and 20.7% neither unprepared nor prepared. One respondent wrote: *“Teachers should be open to such topics first.”*

In the answers to the open-ended questions, remarks were found on teaching a diverse class. A respondent stated: *“Using diverse teaching methods such as cinemeducation [=using film in medical education] that facilitate students and teachers operating outside of their own cultural comfort zone.”* Other suggestions from respondents included: *- “To openly discuss the cultural issues with the students.”*

- “To mix groups of Hungarian and English or German program students.”

- “Gain experiences in cultural differences more directly (interaction between cultures) and then, with more insight be better prepared. For example; last I had a discussion with a patient about being operated between Christmas and new year. No problem she said, I don’t celebrate Christmas.. Good point!“

Respondents indicated relatively high preparedness on the two neutrally formulated items, in which culture was not mentioned explicitly, regarding teaching a diverse classroom; 84.2% answered being somewhat prepared or very prepared to ‘Engage, motivate and encourage participation among all students when teaching’. And 89.7% felt somewhat prepared or very prepared to ‘Create a safe and open atmosphere for all students when teaching’.

**Table V.** Teachers’ opinion of own preparedness to teach a diverse class. Reported in percentage (n)

	Very unprepared	Somewhat unprepared	Neither unprepared nor prepared	Somewhat prepared	Very prepared
Teach students from a range of diverse cultural and religious backgrounds *(missing=5)*	3.4% (33)	11.7% (113)	21.5% (207)	44.7% (430)	18.7% (180)
Examine your own cultural biases that may influence your behaviour as a teacher *(missing=8)*	2.8% (27)	12.5% (120)	20.7% (199)	48.0% (461)	15.9% (153)
Engage, motivate and encourage participation among all students when teaching *(missing=5)*	.8% (8)	3.4% (33)	11.5% (111)	54.0% (520)	30.2% (291)
Create a safe and open atmosphere for all students when teaching *(missing=7)*	.4% (4)	1.9% (18)	8.0% (77)	49.2% (473)	40.5% (389)

### Interest in Receiving Training on Teaching Cultural Competence Topics

Respondents indicated high training needs regarding communication issues. For example, 81% reported to feel somewhat interested or very interested in receiving training on ‘How to address conflict which may arise when there are different cultural views between the care provider and the patient’. 81.5% reported somewhat interested or very interested to receive training on ‘How to teach students to adapt their communication style to respond to the patient’s needs’. And 79.3% were somewhat interested or very interested in receiving training on ‘How to help students explore their own perspectives and values and how these may influence their future practice’. 73% of the respondents were somewhat or very interested in receiving training about ‘Prejudice and discrimination in health care’. A low proportion of 64.2% was somewhat or very interested in receiving training on ‘How to work effectively with interpreters’. ‘How to identify the patient’s language and literacy levels’ scored moderate as well; 67.6% was somewhat to very interested in receiving training on this subject. 69% was somewhat or very interested in receiving training about ‘Disparities in health and health care’.

**Table VI.** Teachers’ interest in receiving training on teaching cultural competence topics. Reported in percentage (n)

Interest in receiving training on:	Very uninterested	Somewhat uninterested	Neither uninterested nor interested	Somewhat interested	Very interested
How to explore the patient’s cultural and religious beliefs that could have an impact on their care *(missing=61)*	1.9% (17)	7.2% (65)	15.3% (139)	46.4% (421)	29.2% (265)
How to explore aspects of the patient’s social context that could have an impact on care *(missing=56)*	2.2.% (20)	6.4% (58)	14.4% (131)	48.7% (444)	28.4% (259)
How to work effectively with interpreters *(missing=58)*	4.4% (40)	9.0% (82)	22.4% (204)	42.2% (384)	22.0% (200)
About prejudice and discrimination in health care *(missing=58)*	2.2% (20)	6.0% (55)	19.2% (175)	43.2% (393)	29.3% (267)
About migrant health (epidemiology, social determinants, access and barriers to care, etc.) *(missing=58)*	1.9% (17)	5.7% (52)	18.4% (167)	46.5% (423)	27.6% (251)
How to address conflict when there are different cultural views between the care provider and the patient *(missing=59)*	1.7% (15)	3.9% (35)	13.5% (123)	45.1% (410)	35.9% (326)
How to prepare students to adapt their communication style to respond to the patient’s needs and capabilities *(missing=63)*	1.2% (11)	4.6% (42)	12.7% (115)	42.8% (387)	38.7% (350)

73.3% was somewhat to very interested in receiving training on ‘How to integrate cultural competence topics in the medical curriculum’. An organisation-wide approach was advocated. As one respondent pointed out: *“Top down encouragement and support, offer regular refresher courses, peer-to-peer coaching.”*

### Interest in Receiving Training on How to Teach a Diverse Classroom

77.7% of respondents reported to be somewhat interested or very interested in receiving training on ‘How to examine your own cultural biases that may influence your behaviour as a teacher’. Some respondents more specifically reported a need for reflection on bias, when asked: What do you think could be done to encourage and support medical teachers to incorporate cultural competence topics into their teaching activities: *“Workshops with focus on cultural differences in teachers own field. Teachers’ own bias in cultural difference”. And yet another wrote: “Initially need facilitated workshops to explore practitioners own cultural bias. Make use of patients’ narrative experiences.”*

80.9% of the respondents were somewhat to very interested in receiving training on ‘How to engage, motivate and encourage participation among all students when teaching’, and 79.8% of respondents were somewhat to very interested in receiving training on ‘How to create a safe and open atmosphere for all students when teaching’.

Respondents suggested:

-“Organize workshops for teachers to learn how to address it in daily teaching activities, like how to create a safe and open sphere in class.”

-“1. Increasing the number of students and teachers from other cultures at the University. 2. Promoting participation of students in classes and promoting the activities of culturally diverse teams.”

-“Learn about engagement of students - be open minded towards different views of health, be open towards good relation with all students independent of cultural background.”

-“Engage faculty with different cultural background.”

**Table VII.** Teachers’ interest in receiving training on teaching a diverse classroom. Reported in % (n)

	Very uninterested	Somewhat uninterested	Neither uninterested nor interested	Somewhat interested	Very interested
Teach students from a range of diverse cultural and religious backgrounds *(missing=62)*	2.0% (18)	6.3% (57)	15.5% (140)	44.8% (406)	31.5% (285)
Examine your own cultural biases that may influence your behaviour as a teacher *(missing=62)*	1.0% (9)	6.4% (58)	14.9% (135)	42.5% (385)	35.2% (319)
Engage, motivate and encourage participation among all students when teaching *(missing=63)*	.8% (7)	3.5% (32)	14.8% (134)	38.1% (345)	42.8% (387)
Create a safe and open atmosphere for all students when teaching *(missing=60)*	.7% (6)	4.1% (37)	15.4% (140)	35.1% (319)	44.7% (406)

### Incorporating Cultural Competence Topics

86% of respondents considered it somewhat or very important to ‘Incorporate cultural competence topics into the medical school curriculum’. It was widely advocated that teachers must become aware first of their training needs. A selection of the answers to the open ended question is presented here below:

• “ *Active discussion on the topic with fellow teachers could make the teachers realize the importance of the problem and to motivate them addressing it.”*
• “ *Somehow encourage their progress from ‘unaware incompetent’ to ‘aware incompetent’- so that they perceive a learning need on cultural competence. Teachers (and care providers) need to become aware that equitable care is not the same as treating all patients similarly.”*
• “ *Make movies from other countries and cultures on discussion of a case - the same clinical situation - from the doctor and patient perspective. I guess it would be quite an eye opener for people watching.”*
• “ *Create room in the curriculum. Discuss topics re cultural competence in an open atmosphere, use cases, use audiovisual means.”*
• “ *Case presentations. Openness to address cultural and language differences in a multi ethnic work/teaching/caring environment, daring to address these issues on a daily basis also among the caretakers and people working there, accept our different ethnicities and backgrounds, also within apparently similar cultural and ethnic groups.”*
• “ *Make use of patients’ narrative experiences.”*
• “ *Train selected, respected clinicians properly first, free them to give presentations, and let them spread the word. This hospital wide campaign should openly be supported by management.”*

## Discussion

The results of this study showed that most respondents agreed that medical education should include training about cultural issues and that students should be assessed about these issues. Additionally, a majority felt prepared to teach a diverse classroom and to engage all students, as well as to create a safe and open atmosphere. Despite their large preparedness to teach cultural issues to students, respondents indicated high training needs regarding communication issues. These results are in line with small-scale studies outside Europe, that also found that medical teachers have a need for cultural competence training ( Berger *et al*. 2014; Lu *et al*. 2014; Rollins et al. 2014). However, in our study, medical teachers are prepared to teach about cultural competencies, but articulate at the same time a need for more training. This suggests that the teachers that participated to our study, are generally the teachers that are interested in cultural competencies and in teaching about diversity. One reason for this may lie in ‘conscious incompetence’ ( Crandall et al., 2003) of the participating teachers who may feel prepared in teaching cultural diversity to diverse students, but may also be aware of where their incompetence still lies or would they assume blind spots within themselves. Given also the low response rate of this study (17 %) it is well possible that the majority of medical teachers in general have never thought about teaching cultural competencies or may even have strong aversions against it.

Our study has several implications for faculty development programs. Firstly, our results imply that medical schools in Europe should make more effort of conceptualizing and framing cultural diversity. We recommend that medical schools and universities take a responsibility in training all health care educators to teach these issues, but also that cultural competence is a compulsory part of the curriculum, including compulsory assessment ( Dogra, 2016), rather than making individual teachers responsible for teaching and assessing. As was reported in our results, it is important to incorporate cultural competence into ongoing educational activities and it needs to be recognised integrally and also covered as a distinct module in the curriculum. The most effective approach to include cultural competence in medical education is in an integral way, top-down, consolidated in institutional policy and supported by deans and heads of departments. As part of the C2ME project, a curriculum scan was performed in eleven European medical faculties, which identified that a key challenge is still to motivate and engage stakeholders (teachers, management etc.) to promote and allocate resources to cultural competence training for teachers and to develop a curriculum that fosters students’ awareness of their own culture without promoting cultural stereotypes ( Sorensen *et al*., 2017a).

Secondly, our results guide the content of future faculty development programs. An important topic that warrants attention is teaching about discrimination, bias and prejudice. We would suggest that to adequately support medical teachers in teaching these sensitive issues to students, while at the same time preserving a safe and inclusive environment within the classroom, training of medical teachers not only needs to include cultural sensitivity and how to recognize unconscious bias, but also should address how to engage in, sustain, and deepen interracial dialogue on race and racism within their medical schools and universities.

Another important topic that may need to be addressed in faculty development programs is the topic of how to address conflict when there are different cultural or religious views between the care provider and the patient. For example, a lack of training in breaking bad news has been put forward to contribute to a practice of withholding the diagnosis to migrant children with cancer (Pergert & Lutzen, 2012). Other studies also found training gaps in teaching cross-cultural issues ( Sturman & Saiepour 2014; Rollins *et al*., 2014), for example, in providing culturally sensitive end-of-life care, dealing with cross-cultural conflicts relating to diagnosis or treatment and eliciting information about the use of folk healers and/or other alternative practitioners.

Another topic is the medical culture itself with its own values, norms and language and which is often taken for granted. Interestingly, respondents in our study reported a higher percentage of agreement, when asked about the perception of patients, looking at health problems through their own cultural lens, than when asked about the perception of care providers looking at the same issue. This may be explained by the professional attitude that care providers take when encountering health problems, when they attempt to deliver optimal personalized medicine and try to perceive the perspective of the patient. Alternatively, care providers may be more inclined to think that only patients look at health issues through a cultural lens. These results may be highlighted in faculty development programs, for example, by reflecting on one’s own taken for granted values about end-of-life care, breaking bad news, or the use of folk healers and how these values may conflict with patients values, but also how to deliver good care despite these conflicting values.

Faculty development programs should also include teaching a diverse classroom. All medical teachers should be able to include all students regardless of their ethnic, cultural or social background in their teaching. It is generally believed that this inclusive teaching may support medical students from ethnic minority backgrounds in their learning process. There is evidence that medical students from ethnic minority background under-perform when compared with those from the ethnic majority (Woolf *et al*. 2011) and qualitative research (Woolf *et al*. 2008) has highlighted the importance of the student-teacher relationship to learning of ethnic minority students and the possible contributory effects of for example stereotyping to their worse performance (Woolf 2008). Medical teachers should increase their awareness to involve all students in their class. A diverse class may be a very highly learning environment, but teachers must be cautious to avoid tokenism, and to promote the inclusion of students with a migrant background, which is not happening per se ( Leyerzapf, 2017).

### Strengths and Limitations

The C2ME project was the first to identify European medical teachers’ opinions on cultural competence. The survey’s questions were designed following the international literature and conjointly discussed, revised and agreed upon by the international team as part of the EU project C2ME. The combination of close-ended items on preparedness and training needs facilitates comparison, as such this study generates knowledge.

Moreover, the open-ended items provide useful insight in some of the motivations of the responses.

Some limitations must also be noticed. The number of respondents as well as the response rate per institution that were invited to participate varied substantially across institutions. Comparison between institutions is therefore not possible: the samples differ in many aspects such as the topics that the respondents teach and their professional backgrounds. Also, there is variance in the social contexts of the different participating countries and how terms and concepts are applied and understood.

As this was a convenience sample with a relatively low response rate, the results cannot easily be generalised to all European medical educators.

Selection bias may have played a role, as it is thinkable that the group of respondents consisted of those teachers who were already interested in teaching cultural competence topics and teaching a diverse class. While these respondents who were generally experienced and interested in teaching these issues expressed a need for training, we suspect that the majority of the European medical teachers would benefit from faculty development programmes.

We would therefore recommend that European medical teachers should be trained in teaching cultural competence topics and in teaching a diverse class. A framework of competencies developed in a recent Delphi study ( Hordijk *et al*., 2018), can be used, which includes competencies that all medical teachers should possess, such as basic knowledge of ethnic and social determinants of health; skills to teach in a non-judgemental way; and skills to engage, motivate, and encourage participation of all students. These faculty development programs should be of practical nature, consisting of small-group discussions, case presentations and consultations with simulation patients. The lecturing part can be handed over by means of an e-learning with short presentations using sheets with key words. We recommend that faculty development programs are assessed as well as evaluated on a regular basis, in order to assure their quality and to adapt them to specific needs of medical teachers.

## Conclusion

This study emphasizes the importance of incorporating cultural issues into the medical curriculum and to train medical teachers according to their needs.

## Take Home Messages

• Most respondents agreed that medical education should include training about cultural issues and that students should be assessed about these issues.
• The majority felt prepared to teach a diverse classroom and to engage all students.
• The most part of respondents were interested in receiving training on teaching cultural competence topics, especially on communication related issues, and, to a somewhat lesser extent, on teaching a diverse class.
• Medical teachers should be trained in teaching cultural competence topics and in teaching a diverse classroom.
• Medical schools in Europe should make more effort of conceptualizing and framing cultural diversity.

## Notes On Contributors

Katja Lanting, MSc, is a Researcher at the Department of Public Health, Amsterdam UMC, location AMC, the Netherlands.

Nisha Dogra, PhD, is Professor of Psychiatry Education and Honorary Consultant in Child and Adolescent Psychiatry at the Greenwood Institute of Child Health, University of Leicester.

Kristin Hendrickx, MD, PhD, is a GP and Professor at the Department of Primary and Interdisciplinary Care, University of Antwerp, Belgium.

Yoga Nathan, PhD, is Senior Lecturer in Medical Education (PBL) at the Graduate Entry Medical School, University of Limerick.

Judith Sim, PhD, is the Course Organiser for the Honours Elective, Social and Ethical Aspects of Medicine of the Biomedical Teaching Organisation (BMTO), Edinburgh Medical School.

Jeanine Suurmond, PhD, is Assistant Professor at the Department of Public Health, Amsterdam UMC, location AMC, the Netherlands.

## Declarations

The author has declared that there are no conflicts of interest.

## Ethics Statement

The study was approved by the Dutch NVMO Ethical Review Board, NERB file number 324.

## External Funding

This article has not had any External Funding
