# Supplementary material for: Culturally Competent in Medical Education – European Medical Teachers’ Self-Reported Preparedness and Training Needs to Teach Cultural Competence Topics and to Teach a Diverse Class
Source: MedEdPublish (2016). 2019 Apr 26;8:98. [Version 1] doi: 10.15694/mep.2019.000098.1 (PMC10712631; doi:10.15694/mep.2019.000098.1)
Supplement: Peer review history

# Reviewer report: Reviewer response for version 1

**DOI:** 10.21956/mep.19808.r29813

**Published:** 2019-06-24

**Authors:** Mary Dankbaar

**Affiliations:** 1 Erasmus University Medical Centre

This review has been migrated. The reviewer awarded 4 stars out of 5 This paper is about educating cultural competence. This is an important topic for medical education, as European countries increasingly constitute of culturally diverse populations. The authors state that in order to improve the generally lower levels of physical and mental health of migrants and ethnic minorities (compared to majority populations), communication barriers, cultural barriers and bias have to be dealt with. The medical curriculum should integrate cultural competences, described as ‘a set of attitudes, knowledge and skills that are necessary for care providers to effectively interact with culturally and ethnically diverse patient populations’. This study was set up to:1. Investigate European medical teachers’ training needs towards teaching cultural competence topics and teaching a diverse class;2. Identify medical teachers’ opinions on cultural competence topics in medical education, their preparedness to teach cultural competence topics and their related training needs.Results of the web-based survey among medical doctors, nurses and public health professionals in 11 European countries showed that most respondents agreed that medical education should include training about cultural issues. A majority felt prepared to teach on cultural competency and in a diverse classroom. However, respondents also state high training needs regarding communication issues.This is a interesting and relevant paper for those interested in medical curriculum design and improving cultural competency. Methods and Results are described clearly. One remark on Methods (second paragraph): ‘Each coordinator forwarded the survey to the relevant staff at their institution’.. what is ‘relevant staff’ in this context? Teacher who presently teach cultural competency? (this is not yet a very dominant part of the curriculum), teachers who teach communication skills, or other subjects? As this was not explained or operationalized, this choice may have varied strongly per county, influencing the results. This should be mentioned in Limitations.In addition, the question is to what extent the authors believe that social desirability played a role in answering the questions? Will the respondents vote in favor of including cultural issues in their curriculum, even when this means their own subject will be reduced in teaching hours? A critical discussion of these answers would have given the Discussion more depth. There is a good Discussion relating the results to previous studies, and discussing implications for the curriculum. In the strengths and limitations, strong points and possible weaknesses of the study, such as a possible selection bias, are discussed. In conclusion, this article is certainly a useful contribution to the field.

Reviewer Expertise:

NA

No decision status is available

# Reviewer report: Reviewer response for version 1

**DOI:** 10.21956/mep.19808.r29815

**Published:** 2019-05-13

**Authors:** Balakrishnan Nair

**Affiliations:** 1 Centre for Medical Professional Development and University of Newcastle

This review has been migrated. The reviewer awarded 4 stars out of 5 Thanks for asking me to review this very interesting paper. We live in an era where patients , learners and teachers are from diverse groups. Patients and carers look at health issues through their own cultural lenses . As the authors state migrants have more health issues ; unfortunately they have more unmet needs. So this study looking at the attitude and insight of teachers into this area is worthwhile. The only concern is the low response rate . However it is reassuring to note that the majority of respondents agreed this is an important area needing more action , including assessment. I am keen to see translation of this research into action .

Reviewer Expertise:

NA

No decision status is available

# Reviewer report: Reviewer response for version 1

**DOI:** 10.21956/mep.19808.r29811

**Published:** 2019-05-11

**Authors:** Trevor Gibbs

**Affiliations:** 1 AMEE

This review has been migrated. The reviewer awarded 4 stars out of 5 An interesting paper written about an important subject. I enjoyed reading this well written paper and certainly learned from the introduction section and the very useful set of references. i was not really surprised by the findings of the survey, although I would have hoped for a better response rate, which might have biased this paper somewhat.This paper provides very important information for those of us who believe that cultural competence should be present in all health professions curricula and as such would be happy to recommend it to all curriculum planners.Of course the next major step is to translate these findings into action- not an easy task

Reviewer Expertise:

NA

No decision status is available

# Reviewer report: Reviewer response for version 1

**DOI:** 10.21956/mep.19808.r29812

**Published:** 2019-04-29

**Authors:** P Ravi Shankar

**Affiliations:** 1 American International Medical University

This review has been migrated. The reviewer awarded 5 stars out of 5 Thank you for the opportunity to review this interesting manuscript. Cultural competence is becoming increasingly important and doctors should be able to provide care to diverse populations. Teaching cultural competence can be challenging as mentioned in the article. The authors have described their methodology very well. The Results and Discussion have been clearly described. They also have highlighted the strengths and limitations of their study. Learning about and being aware of specific cultural issues while treating a particular patient can be challenging. The issues to be aware of are vast and challenging. In the future, machine learning algorithms could help doctors. This is an important study conducted in multiple European countries. The list of references is comprehensive. All medical educators will find this study of interest.

Reviewer Expertise:

NA

No decision status is available

# Reviewer report: Reviewer response for version 1

**DOI:** 10.21956/mep.19808.r29814

**Published:** 2019-04-26

**Authors:** Felix Silwimba

**Affiliations:** 1 University of Lusaka

This review has been migrated. The reviewer awarded 5 stars out of 5 Cultural competence in medical education for the educators and the medical students. This is a very important item in the provision of quality medical care. This study is relevant and applicable to all regions of the world. They are so many divergent micro cultures within regions, countries and sub regions that influence the health of the people. I agree medical educators should be the forefront of being culturally aware and educated in order to pass on the knowledge to medical students. I recommend this article.

Reviewer Expertise:

NA

No decision status is available
